# Achieving long-term goals through early personalized management of schizophrenia: expert opinion on the role of a new fast-onset long-acting injectable antipsychotic

**DOI:** 10.1186/s12991-022-00430-1

**Published:** 2023-01-17

**Authors:** Antonio Vita, Andrea Fagiolini, Giuseppe Maina, Claudio Mencacci, Edoardo Spina, Silvana Galderisi

**Affiliations:** 1 Department of Mental Health and Addiction Services, Spedali Civili of Brescia, University of Brescia, Brescia, Italy; 2 Department of Psychiatry, University of Siena, Siena, Italy; 3 Department of Neuroscience Rita Levi Montalcini, University of Turin, Turin, Italy; 4 Neuroscience Mental Health ASST FBF-Sacco, Milan, Italy; 5 Department of Clinical and Experimental Medicine, University of Messina, Messina, Italy; 6 Department of Psychiatry, University of Campania Luigi Vanvitelli, Naples, Italy

**Keywords:** Schizophrenia, Long-acting injectable, Risperidone ISM^®^, Therapeutic goals

## Abstract

Definition of an appropriate and personalized treatment plan focused on long-term outcomes is crucial in the management of schizophrenia. Following review of the literature, a panel of six leading psychiatrists discussed the importance of clear and shared long-term goals when initiating antipsychotic treatment in light of their clinical experience. The importance of establishing shared and progressive treatment objectives was stressed, which should be tailored based on the patient’s characteristics, goals, and preferences. Consensus emerged on the key role that therapeutic alliance and patient empowerment play throughout the course of treatment. Reduction in symptoms in the acute phase along with good efficacy and tolerability in the maintenance phase emerged as essential features of a therapy that can favor achievement of long-term outcomes. Long-acting injectable (LAI) antipsychotics enhance adherence to treatment compared to oral formulations and have been shown to be effective in the maintenance phase. Currently available LAIs are characterized by a delayed onset of action and require a loading dose or oral supplementation to achieve therapeutic concentrations. Risperidone ISM^®^ is a novel LAI antipsychotic with fast and sustained release of antipsychotic, reaching therapeutic plasma levels within a few hours after administration without oral supplementation or loading doses. Risperidone ISM^®^ has been shown to rapidly control symptoms in patients with an acute exacerbation of schizophrenia and to be effective and well tolerated as maintenance treatment irrespective of the severity of initial symptoms. It thus represents a valuable and novel therapeutic option in management of schizophrenia.

## Introduction

Schizophrenia is often a chronic and disabling disorder, characterized by clinical relapses and frequent unfavorable progression, with a profound negative impact on psychosocial functioning, relationships, and cognitive functions [1–3]. Relapse can be distressing for patients as well as families and caregivers, thus threatening the ability to live an independent life, with an increased risk of hospitalization, burden of care, and treatment costs [1, 4]. Relapse is also associated with neurobiological damage and the development of treatment resistance, even in previously responsive patients [5].

Antipsychotics are the mainstay in treatment of schizophrenia and significantly improve treatment outcomes, quality of life, and life expectancy in all stages of the disease [1, 6–8]. An extended discussion of different therapeutic options is beyond the scope of this article, although it is established that continuous long-term antipsychotic treatment is essential for relapse prevention, which is a key treatment goal in schizophrenia [9]. Shared decision-making, in which physicians provide information to patients and patients provide information on their goals and preferences, is receiving increasing attention in the management of patients with schizophrenia and choice of therapy [10, 11]. This joint collaboration is held to be a valid strategy to maximize recovery and improve both outcomes and adherence to treatment through development of a personalized treatment strategy [12]. In fact, continuous treatment with antipsychotic drugs effectively reduces relapses and hospitalization rates. Indeed, in case of treatment discontinuation, the risk of relapses at 1 year is almost 3 times higher, thus demonstrating the benefits of long-term antipsychotic treatment, despite the possible occurrence of side effects [13]. Early treatment represents another key aspect in schizophrenia, as an increased duration of untreated psychosis (DUP) is associated with poorer long-term clinical and functional outcomes and worse quality of life [14]. On the other hand, continuity of treatment from the early stages of the illness can help improve long-term outcomes [15–17].

In this context, therapeutic interventions that enhance adherence to treatment, such as long-acting injectable (LAI) antipsychotics, represent a valuable option to increase treatment persistence and achieve lasting therapeutic goals [18–20]. LAI antipsychotics have been shown to improve several clinical outcomes, including relapse rate and number and duration of hospitalizations, as well as survival and life expectancy [8, 21–23]. LAIs have also been suggested to prevent white matter deterioration, which is associated with progression of illness [24]. Moreover, the use of LAI antipsychotics may allow for successful rehabilitation and social reintegration by facilitating psychosocial interventions [1, 6–8].

Even if recommended as the treatment of choice in the long-term management of schizophrenia, the latency in reaching therapeutic plasma concentrations of the available LAI antipsychotics imposes the need for either supplementation with oral antipsychotics or a loading dose, thus limiting their use in all phases of the disease [25–27]. Risperidone ISM^®^ is a novel LAI antipsychotic that was developed to overcome these limitations, thanks to its innovative technology that ensures fast and sustained release of the active principle with control of symptoms in the absence of oral supplementation or loading dose [28–31].

A panel of six leading psychiatrists and experts in the treatment of schizophrenia evaluated the potential place in therapy of Risperidone ISM^®^ by reviewing the relevant literature on this new compound in the light of their clinical experience. The resulting expert opinion aims to define a new therapeutic approach aimed at reaching comprehensive long-term objectives since the start of treatment.

## Methods

Six Italian experts in psychiatry and schizophrenia treatment reviewed the published literature on relevant aspects of schizophrenia patient management, with an emphasis on the definition of long-term treatment objectives and strategies to maximize treatment success. The critical appraisal of the scientific evidence was supported by the vast clinical experience of the authors.

### Establishing long-term objectives at the beginning

The therapeutic path of patients with schizophrenia includes both short- and long-term goals. Thanks to the advent of new antipsychotic treatments and the introduction of effective psychosocial interventions, therapeutic objectives have evolved over time and shifted from control of acute psychotic and behavioral symptoms to long-term goals such as improving psychosocial functioning and quality of life with the aim of attaining both clinical and personal recovery [32]. Nonetheless, increasing adherence to antipsychotic treatment and preventing relapses remain key treatment goals [2, 3, 33]. In fact, relapses determine an unfavorable progression of illness, with deep impact on social and cognitive functions [34]. Relapses are distressing for patients and caregivers, disrupt the trajectory of the patient’s recovery, and increase the burden and costs of care [1, 4]. The panel acknowledged the paramount importance of reducing the risk of relapse, referring that relapses undermine trust on the possibility to achieve therapeutic success for patients, their families, and psychiatrists, with detrimental effects on long-term outcomes. Thus, ensuring adherence to antipsychotic treatment is crucial in preventing relapses, and represents a major challenge in the management of patients with schizophrenia [35–37]. Non-adherence increases the risk of relapse by 5 times and 40–60% of patients treated with an oral antipsychotic are partially or totally non-adherent to therapy, with treatment discontinuation rates ranging from 26 to 44% [38–40]. In this regard, LAI antipsychotics can favor greater adherence and improve persistence on therapy compared to oral formulations [21, 23]. Reducing the DUP through timely interventions also greatly improves the possibility of achieving a favorable therapeutic outcome [14, 34]. The experts agreed on the benefits of LAI formulations in terms of increased continuity of care, especially if such therapies are introduced early during the course of illness [34, 41, 42].

Even in subjects with acute exacerbations of symptoms, the need to develop a treatment plan that is focused not only on rapid symptom control but also on long-term goals was recognized by the panel. Achieving short-term objectives rapidly allows the physician to promptly initiate discussion on long-term objectives, which should be based on the individual characteristics and realistic goals, and shared with the patient [12]. In order to favorably affect long-term clinical outcomes, the experts agreed on the importance of providing a treatment that can be used in the acute phase to rapidly control symptoms, but which is also effective and well tolerated as maintenance therapy. It was reasoned that fast management of symptoms can improve overall prognosis by preventing further relapses and allowing focus on non-pharmacological interventions, thus establishing an integrated treatment which is essential to achieve recovery [34]. Moreover, it was highlighted that the patient’s care path should be a continuum across the different treatment settings with the aim of achieving well-defined and shared therapeutic goals.

In agreement with recently published international guidelines [43, 44], the recommendation made by the panel was to initiate treatment with a LAI shortly after stabilization following an acute psychotic exacerbation and, in case of a first hospitalization, before the patient’s discharge. A LAI that does not require loading dose or oral supplementation has the potential advantage of ensuring sustained therapeutic levels of active drug from the first administration, which overcomes the need for adherence to oral therapies and for prolonged hospitalizations until the patient receives a second injection.

The experts also recommended that the therapeutic approach should be personalized according to the individual characteristics of the patient. Personalization should refer to the establishment of long-term goals, which may differ depending upon the patient’s gender, age, clinical characteristics, and psychopathological condition, as well as level of cognitive impairment, social and psychosocial needs, school and work opportunities and performance, and tolerability to a given treatment [12, 45]. The authors also acknowledged that despite numerous advantages associated with the use of LAIs, limited availability of healthcare, economic, and technological resources for mental health services may influence their prescription.

### The role of therapeutic alliance and patient empowerment in an effective pathway of care

Therapeutic alliance and patient empowerment are essential aspects of an integrated treatment approach focused on the patient’s expectations and needs. According to the panel, without a well-established therapeutic alliance between all those involved, including the patient, physician, and caregiver, it is not possible to achieve long-term clinical outcomes. The therapeutic alliance can positively affect the patient’s attitude towards treatment, thus reducing potential negative perceptions and improving adherence to therapy [46–49]. In fact, even for patients who experience an involuntary admission, a positive experience during the hospitalization and establishing a good relationship with the treating clinician could lead to more easily accepting subsequent therapies [50]. The experts highlighted that the sharing of information between the patient and psychiatrist is the cornerstone of an effective therapeutic alliance. This should follow the principle of warranting dignity and respect to the patient, who should receive clear information from the treating clinician on the available therapeutic options and be involved in the decision-making process. This was regarded by the panel as a key aspect to enhance treatment acceptance and subsequent adherence [41]. Moreover, patients should also be involved in setting feasible and meaningful therapeutic goals, depending upon their individual characteristics and objectives. The experts recommended establishing progressive and realistically achievable goals in order to prevent potential frustration associated with failure.

Reciprocal trust between the patient and physician is built over time and needs to be continuously renewed and reconfirmed. The therapeutic alliance can also be sustained by the establishment of a treatment perspective even in the most complicated phases of the disease. To reinforce this alliance, it is important for the clinician to embrace empathetic attitudes, refraining from paternalistic and only assertive behaviors. In order to make the patient feel as a “person” and not as a “schizophrenic patient”, the panel emphasized the importance of listening to the patient’s needs and concerns, rather than focusing solely on clinical aspects like symptom assessment and remission. An effective therapeutic alliance stems from a cooperative relationship between different healthcare professionals interacting with each other, as well as with the patient and his/her family or caregiver [51, 52]. However, the available literature data show that patients and their caregivers are often poorly involved in the decision-making process [53]. A significant contribution to sustaining the alliance can be made by the patient’s family. The caregiver can provide valuable support for both the patient and the physician by promoting adherence to treatment and ensuring continuity of care, while an unfavorable experience can undermine the alliance. However, the experts emphasized that it is crucial to discuss and agree with the patient about the direct involvement of the family in the therapeutic path. In fact, in some cases, especially for outpatients or discharged patients, the family can represent either a resource for the patient’s well-being or a possible cause of psychological distress.

The experts recognized that the use of LAI antipsychotics could improve the relationship between the physician and patient and help maintain the therapeutic alliance thanks to improved adherence and reduced rate of relapse [21, 23]. In fact, in their experience, another deleterious consequence of relapses is that they undermine the patients’ trust in their physicians, as well as in their families and caregivers. Moreover, the use of a LAI can improve the relationship between patients and psychiatrists by increasing the time available during visits to discuss other relevant aspects for the treatment path, without focusing only on adherence [41, 54, 55]. The panel also referred that many patients perceive this formulation as a convenient and desirable option, which is in agreement with studies showing that treatment acceptance with LAIs was highest among patients who are currently or were already treated with this therapeutic option [56].

Despite evidence that LAI therapy is efficacious in controlling symptoms and preventing relapses of schizophrenia, LAI antipsychotics are underutilized mainly because of attitudinal barriers from psychiatrists, rather than skepticism from patients [56–58]. The panel agreed that psychiatrists should consider LAIs the choice of treatment not only for patients with a history of non-adherence and multiple relapses, but also for those at an early stage of illness [59]. The physician’s attitudes towards LAIs have been shown to influence how this therapeutic alternative is presented to patients, the treatment acceptance, and effective prescription [60]. Barriers to the use of LAIs include lack of knowledge, fear of damaging the therapeutic alliance, perception of disease worsening, lack in flexibility, route of administration and dosages, and delayed therapeutic onset [60]. Risperidone ISM^®^ bypasses the need for psychiatrists to negotiate the administration of a second injection or oral supplementation, which can, therefore, facilitate discussion with patients about this therapeutic option and treatment acceptance.

Patient empowerment leads to greater patient autonomy, both in decision-making and in terms of self-management. Its importance in helping to achieve long-term treatment goals was acknowledged by the panel [61, 62]. Patient empowerment is enabled by shared information and close collaboration with the physician, who plays a fundamental role in eliciting disease awareness and patient’s involvement in the therapeutic plan [63]. Based on their clinical experience, the experts confirmed that personalized therapies could promote empowerment by increasing self-esteem and independence. The panel acknowledged that improving patient empowerment is applicable, albeit to different extents, to every patient with schizophrenia, regardless of previous history or phase of illness. To this end, not only healthcare professionals but also the patient’s family and social environment should play a significant role and be actively involved. LAI formulations can be hypothesized to indirectly favor patient empowerment. Risperidone ISM^®^, thanks to its fast and sustained control of symptoms, without need of oral supplementation or loading dose, may facilitate patient empowerment by increasing treatment adherence and continuity, allowing the medical team to focus on the patient’s involvement and on achieving personal goals [34].

### Clinical significance of a new LAI with fast onset

Risperidone ISM^®^ is a new LAI antipsychotic that has recently received marketing authorization approval by the European Medicines Agency for treatment of adult patients with schizophrenia. The tolerability and efficacy of oral risperidone and its active pharmacological compound have been well established. Risperidone ISM^®^ combines the well-known clinical efficacy of risperidone with an innovative mechanism of release based on the ISM^®^ (in situ microparticles) technology. The drug is reconstituted in an injectable suspension that after intramuscular injection at either the gluteal or deltoid muscles precipitates in situ to form a solid or semisolid matrix. Risperidone ISM^®^ reaches therapeutic plasma concentrations within the first few hours following administration and provides a sustained release of the drug throughout a 4-week period without any prior loading dose regimen or supplementation with oral risperidone [64].

Thanks to this innovative release technology, Risperidone ISM^®^ induces a fast reduction in symptoms from day 8 after the first injection in patients with acute schizophrenia [29]. According to the panel, the rapid onset of action is an undeniable advantage in this phase of illness, thus representing a good option for treatment of hospitalized patients. Furthermore, the absence of oral supplementation represents an additional clinical advantage of Risperidone ISM^®^. In fact, oral supplementation can be considered as a limitation that can hinder the prescription of LAIs in sub-acute phases, when many problems with adherence occur.

The panel welcomed the possibility to deploy this novel and improved LAI formulation based on risperidone, which is particularly effective in the management of positive symptoms that are prevalent in the acute phase of the disease. In fact, the currently available risperidone LAI has several limitations, such as biweekly administration, oral supplementation, and need for cold chain [65, 66], which may limit its use in clinical practice. While a detailed comparison between the available therapeutic options is beyond the scope of this article, Table 1 summarizes the main features of second-generation LAI antipsychotics. In the case of Risperidone ISM^®^, therapeutic plasma levels are reached within the first hours after injection, achieving steady state concentrations after the first dose that are maintained within the therapeutic range throughout the treatment period [64], without a loading dose or oral supplementation, unlike other currently available LAIs. In fact, for paliperidone palmitate LAI, following the first intramuscular injection, median plasma concentrations gradually decrease, with the need for a second injection 7 days later to achieve therapeutic concentrations [67, 68]. Treatment initiation with aripiprazole monthly requires either oral aripiprazole supplementation for 14 days or a double injection on the first day followed by a single supplementary oral dose [69, 70]. For these reasons, the panel acknowledged the unique pharmacokinetic profile of Risperidone ISM^®^, which may be defined as a “fast-LAI”.

**Table 1:** Second-generation LAI antipsychotics: comparative profile [25, 26, 71]

	Aripiprazole monohydrate	Olanzapine pamoate	Paliperidone palmitate once monthly^a^	Paliperidone palmitate every 3 months^b^	Risperidone microspheres	Risperidone ISM^®^
Refrigeration	No	No	No	No	Yes	No
Approved injection site	Deltoid or gluteal muscle	Gluteal muscle	Deltoid or gluteal muscle	Deltoid or gluteal muscle	Deltoid or gluteal muscle	Deltoid or gluteal muscle
Dosage forms/strengths	Vial kits: 300 mg, 400 mg	Vial kits: 210 mg, 300 mg, 405 mg	Injectable suspension: 39 mg, 78 mg, 117 mg, 156 mg, 234 mg	Injectable suspension: 273 mg, 410 mg, 546 mg, 819 mg	Vial kits: 12.5 mg, 25 mg, 37.5 mg, 50 mg	Vial kits: 75 mg, 100 mg
Oral supplementation	-Single injection: 10–20 mg for 2 weeks after the initial injection -Double injection: 20 mg of oral aripiprazole on day 1	No	No	No	For 3 weeks after the initial injection	No
Dose interval	4 weeks	2 or 4 weeks	Monthly	Every 3 months	2 weeks	4 weeks
Starting dose	-Single injection 400 mg -Double injection on day 1 400 mg (deltoid) + 400 mg (gluteal)	210 mg/2 weeks 300 mg/2 weeks 405 mg/4 weeks	Double injection 234 mg on day 1 and 156 mg on day 8 (deltoid)	273, 410, 546, 819 mg (3.5 times the last dose of the one-monthly formulation)	25 mg	75–100 mg
Maintenance dose	400 mg (300–400 mg)	Up to 300 mg/2 weeks	117 mg (39–234 mg)	273–819 mg	25 mg (25–50 mg)	75 mg
Time to peak	5–7 days	4 days	13 days	30–33 days	4–6 weeks	48 h
Time to steady state	300 mg: 3–4 months 400 mg: 4–8 months	3 months	7–11 months	Continues steady state at equivalent dose	1.5–2 months	4 weeks
Post-injection observation or monitoring	No	Yes or at least 3 h	No	No	No	No

^a^The strengths expressed as 39, 78, 117, 156, and 234 mg paliperidone palmitate equate to 25, 50, 75, 100, and 150 mg paliperidone, respectively

^b^The strengths expressed as 273, 410, 546, and 819 mg paliperidone palmitate equate to 175, 263, 350, and 525 mg paliperidone, respectively

Furthermore, Risperidone ISM^®^ has been shown to be effective, safe, and well tolerated as maintenance treatment in patients affected by schizophrenia in a 52 week open label study with reduction in symptoms irrespective of their initial severity [30]. Although the criteria to define a relapse of schizophrenia vary in the literature, the global relapse rate in this study (10.7%) was lower than relapse rates (24%) reported for different antipsychotics during maintenance treatment of schizophrenia, as reported in a recent meta-analysis [13]. Both dosages of Risperidone ISM^®^ were well tolerated, with a high rate of study completion (74.9%) and only 7 (3.2%) patients discontinuing treatment because of drug-related adverse events. The panel considered Risperidone ISM^®^ as a promising therapeutic option also in consideration of its long-term efficacy and tolerability profile. In particular, the rate of hyperprolactinemia and related symptoms were low in this study, with only 2 of 215 patients discontinuing treatment for adverse events related to hyperprolactinemia. While confirmation in real-world settings is needed, these results nonetheless compare favorably with the experts’ clinical experience with patients being treated with other risperidone-based therapies, both oral and LAI.

## Conclusions

The achievement of long-term goals in patients with schizophrenia relies on early and continued use of effective and well-tolerated pharmacological treatment, prevention of relapses, and integration with evidence-based psychosocial interventions. It is crucial to establish a shared and personalized treatment plan that can improve long-term clinical outcomes starting from the initial phases of the disease. Ideally, this should be facilitated by a single pharmacological therapy that can be used in both the acute and maintenance phases and can favor good adherence. LAI antipsychotics can improve several clinical and non-clinical outcomes; however, the lag period in reaching therapeutic concentrations during the initiation phase and the delayed onset of action limits their use in the hospital setting. Risperidone ISM^®^ is a new LAI antipsychotic with a fast onset of action without the need for oral supplementation or loading dose, which has shown good efficacy, safety, and tolerability profile in the long term, in all types of patients with schizophrenia. The absence of oral co-administration or loading dose may promote acceptance of therapy and support the therapeutic alliance starting with the acute phase, thereby helping to promote and achieve long-term treatment outcomes and personalized objectives. The experts agreed that, given the unique pharmacokinetic profile of Risperidone ISM^®^ and the fast and sustained symptom control demonstrated during its clinical development program, this new drug could help illness management in all phases of schizophrenia, thereby enhancing the possibility of achieving stable clinical recovery.

## Data Availability

Not applicable.

## Abbreviations

LAI: Long-acting injectable
ISM^®^: In situ microparticles
